# Supplementary material for: Mathematical modeling the order of driver gene mutations in colorectal cancer
Source: PLoS Comput Biol. 2023 Jun 27;19(6):e1011225. doi: 10.1371/journal.pcbi.1011225 (PMC10332632; doi:10.1371/journal.pcbi.1011225)
Supplement: S1 Text — Fig A: All pathways of gene mutations for the case (A) in colorectal cancer. Fig B: All pathways of gene mutations for the case (B) in colorectal cancer. Fig C: All pathways of gene mutations for the case (C) in colorectal cancer. Fig D: All pathways of gene mutations for the case (D) in colorectal cancer. Fig E: All pathways of gene mutations for the case (E) in colorectal cancer. Fig F: All pathways of gene mutations for the case (F) in colorectal cancer. (PDF) [file pcbi.1011225.s001.pdf]

S1 Text
Mathematical modeling the order of driver gene mutations in colorectal cancer

by Lingling Li, Yulu Hu, Yunshan Xu, Sanyi Tang

S1 Supplementary Figures

For the sequence $KRAS \longrightarrow APC \longrightarrow TP53$ (A), the detailed gene alterations are depicted in Fig A. Because the net growth rate (λ_i) remains the same whether APC^+ comes first or $TP^{53+/-}$ comes first, only five models are showed based on the clonal expansion of cells in Fig A. In fact, there are eight pathways for the order with $KRAS \longrightarrow APC \longrightarrow TP53$, and Figs A3-A5 represent two pathways, respectively. It is similar for other orders. The detailed gene alterations are depicted in Figs B–F for the sequences $KRAS \longrightarrow TP53 \longrightarrow APC$ (B), $APC \longrightarrow TP53 \longrightarrow KRAS$ (C), $APC \longrightarrow KRAS \longrightarrow TP53$ (D), $TP53 \longrightarrow APC \longrightarrow KRAS$ (E) and $TP53 \longrightarrow KRAS \longrightarrow APC$ (F), respectively.

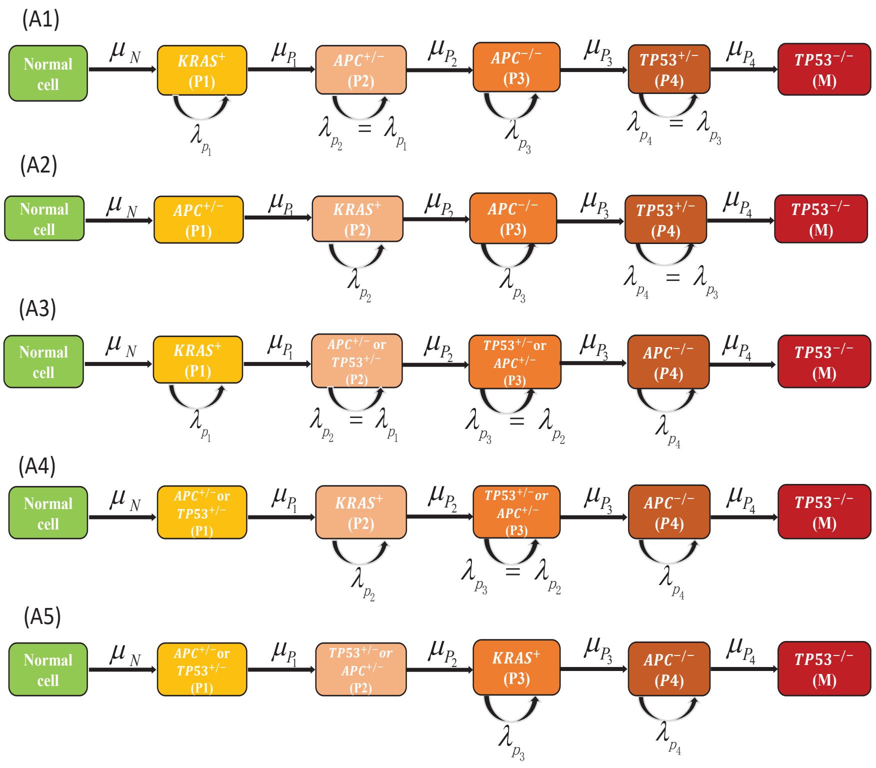

Fig A. All pathways of gene mutations for the case (A) in colorectal cancer.

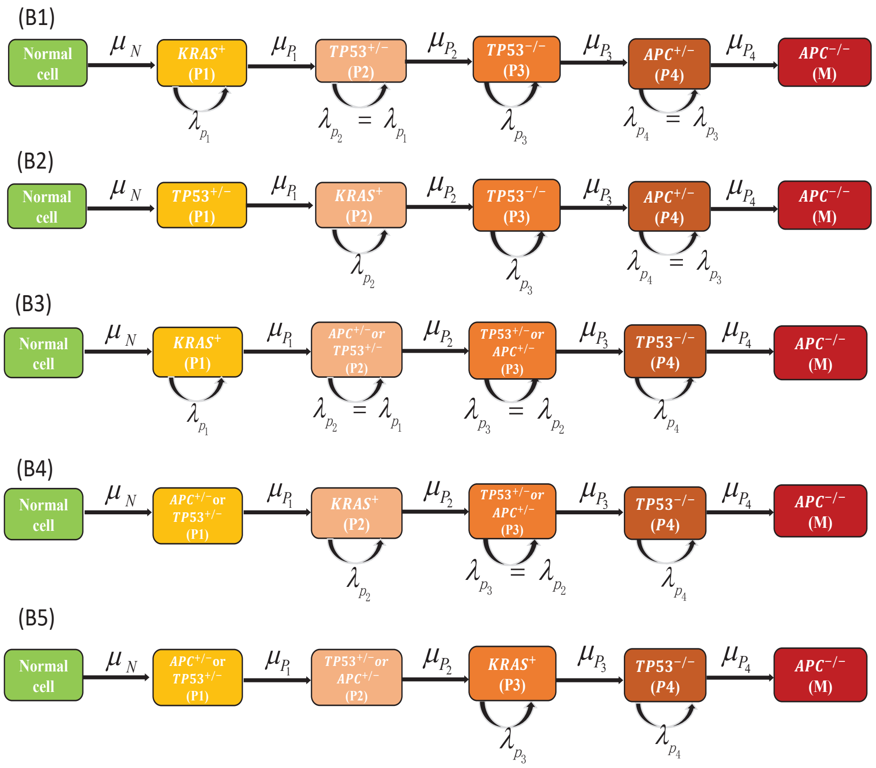

Fig B. All pathways of gene mutations for the case (B) in colorectal cancer.

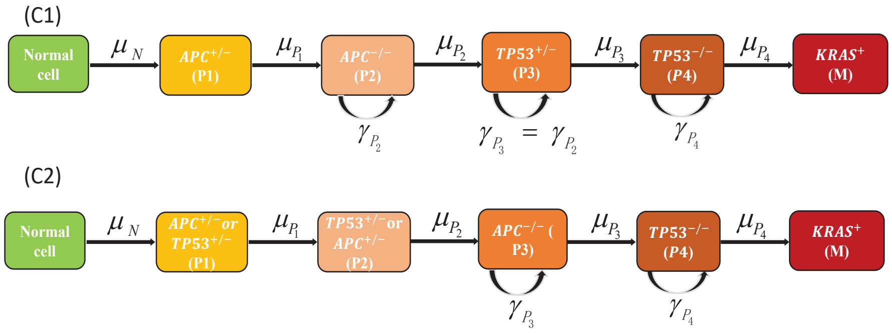

Fig C. All pathways of gene mutations for the case (C) in colorectal cancer.

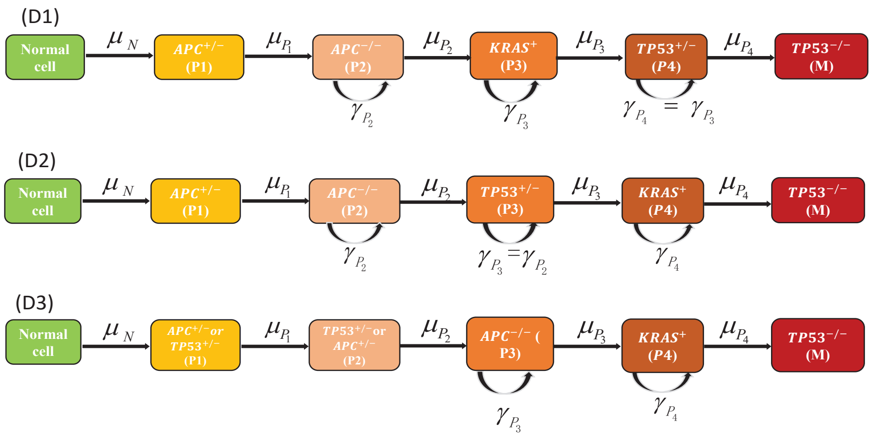

Fig D. All pathways of gene mutations for the case (D) in colorectal cancer.

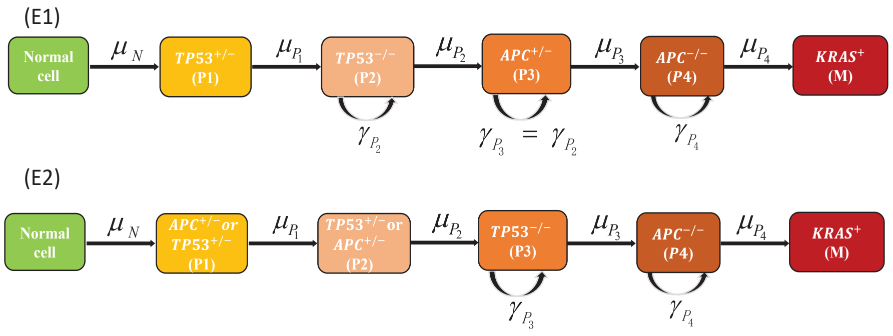

Fig E. All pathways of gene mutations for the case (E) in colorectal cancer.

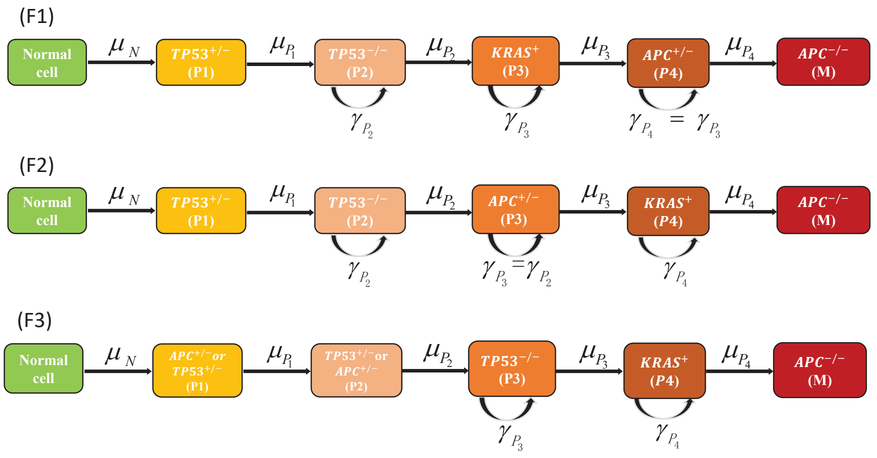

Fig F. All pathways of gene mutations for the case (F) in colorectal cancer.

S2 Mathematical derivation

The derivation of approximate solution

For the model with five stages in Fig 1 of the text, we define the following probability generating function

$$\begin{aligned}\Psi(x_1,x_2,x_3,x_4,y;0,t) = \sum_{i_1,i_2,i_3,i_4,j} & prob\{P_1(t)=i_1,P_2(t)=i_2,P_3(t)=i_3,\\ & P_4(t)=i_4,M(t)=j|P_1(0)=0,P_2(0)=0,P_3(0)\\ & =0,P_4(0)=0,M(0)=0\}x_1^{i_1}x_2^{i_2}x_3^{i_3}x_4^{i_4}.\end{aligned} \tag{S1}$$

Let $P_{i_1,i_2,i_3,i_4,j}(t) = prob\{P_1(t)=i_1,P_2(t)=i_2,P_3(t)=i_3,P_4(t)=i_4,M(t)=j|P_1(0)=0,P_2(0)=0,P_3(0)=0,P_4(0)=0,M(0)=0\}$, which satisfies the following Kolmogorov forward differential equation [1]

$$\begin{aligned}\frac{d(P_{i_1,i_2,i_3,i_4,j}(t))}{dt} = & -(N\mu_N + i_1(\alpha_{p_1}+\beta_{p_1}+\mu_{p_1})P_{i_1,i_2,i_3,i_4,j}(t)\\ & +((i_1-1)\alpha_{p_1}+N\mu_N)P_{i_1-1,i_2,i_3,i_4,j}(t)\\ & +(i_1+1)\beta_{p_1}P_{i_1+1,i_2,i_3,i_4,j}(t)\\ & +i_1\mu_{p_1}P_{i_1,i_2-1,i_3,i_4,j}(t)\\ & -i_2(\alpha_{p_2}+\beta_{p_2}+\mu_{p_2})P_{i_1,i_2,i_3,i_4,j}(t)\\ & +(i_2-1)\alpha_{p_2}P_{i_1,i_2-1,i_3,i_4,j}(t)\\ & +(i_2+1)\beta_{p_2}P_{i_1,i_2+1,i_3,i_4,j}(t)\\ & +i_2\mu_{p_2}P_{i_1,i_2,i_3-1,i_4,j}(t)\\ & -i_3(\alpha_{p_3}+\beta_{p_3}+\mu_{p_3})P_{i_1,i_2,i_3,i_4,j}(t)\\ & +(i_3-1)\alpha_{p_3}P_{i_1,i_2,i_3-1,i_4,j}(t)\\ & +(i_3+1)\beta_{p_3}P_{i_1,i_2,i_3+1,i_4,j}(t)\\ & +i_3\mu_{p_3}P_{i_1,i_2,i_3,i_4-1,j}(t)\\ & -i_4(\alpha_{p_4}+\beta_{p_4}+\mu_{p_4})P_{i_1,i_2,i_3,i_4,j}(t)\\ & +(i_4-1)\alpha_{p_4}P_{i_1,i_2,i_3,i_4-1,j}(t)\\ & +(i_4+1)\beta_{p_4}P_{i_1,i_2,i_3,i_4+1,j}(t)\\ & +i_4\mu_{p_4}P_{i_1,i_2,i_3,i_4,j-1}(t).\end{aligned} \tag{S2}$$

Let's multiply both sides of above equation by $x_1^{i_1}x_2^{i_2}x_3^{i_3}x_4^{i_4}$, we can obtain,

$$\begin{aligned}\frac{d\Psi(x_1,x_2,x_3,x_4,y;0,t)}{dt} = & (x_1-1)\mu_N N\Psi(x_1,x_2,x_3,x_4,y;0,t) + [\mu_{p_1}x_1x_2 + \alpha_{p_1}x_1^2\\ & +\beta_{p_1} - (\alpha_{p_1}+\beta_{p_1}+\mu_{p_1})x_1]\frac{d\Psi(x_1,x_2,x_3,x_4,y;0,t)}{dx_1}\\ & +[\mu_{p_2}x_2x_3 + \alpha_{p_2}x_2^2 + \beta_{p_2} - (\alpha_{p_2}+\beta_{p_2}+\mu_{p_2})x_2]\frac{d\Psi(x_1,x_2,x_3,x_4,y;0,t)}{dx_2}\\ & +[\mu_{p_3}x_3x_4 + \alpha_{p_3}x_3^2 + \beta_{p_3} - (\alpha_{p_3}+\beta_{p_3}+\mu_{p_3})x_3]\frac{d\Psi(x_1,x_2,x_3,x_4,y;0,t)}{dx_3}\\ & +[\mu_{p_4}x_4y + \alpha_{p_4}x_4^2 + \beta_{p_4} - (\alpha_{p_4}+\beta_{p_4}+\mu_{p_4})x_4]\frac{d\Psi(x_1,x_2,x_3,x_4,y;0,t)}{dx_4}.\end{aligned} \tag{S3}$$

By the definition of the probability generating function (S1), we found that $\frac{d\Psi(x_1,x_2,x_3,x_4,y;0,t)}{dx_i}$ is the expectation at time t for the number of the premalignant cells, Pi. From the equation (S3), we get

$$\begin{cases}\frac{dE[P_1(t)]}{dt} = \lambda_{p_1}E[P_1(t)] + \mu_N N,\\ \frac{dE[P_2(t)]}{dt} = \lambda_{p_2}E[P_2(t)] + \mu_{p_1}E[P_1(t)],\\ \frac{dE[P_3(t)]}{dt} = \lambda_{p_3}E[P_3(t)] + \mu_{p_2}E[P_2(t)],\\ \frac{dE[P_4(t)]}{dt} = \lambda_{p_4}E[P_4(t)] + \mu_{p_3}E[P_3(t)],\end{cases} \tag{S4}$$

where $\lambda_{p_i} = \alpha_{p_i} - \beta_{p_i}$.

The approximate solution of hazard function can be given by

$$\begin{aligned} h(t) \approx & \tfrac{N\mu_N\mu_{p_1}\mu_{p_2}\mu_{p_3}\mu_{p_4}}{\lambda_{p_1}^2\lambda_{p_3}^2(\lambda_{p_3}-\lambda_{p_1})^3}[(\lambda_{p_1}^2\lambda_{p_3}(\lambda_{p_3}-\lambda_{p_1})(t-T_{lag})+\lambda_{p_1}^2(\lambda_{p_1}-3\lambda_{p_3})) \\ & exp(\lambda_{p_3}(t-T_{lag}))+(\lambda_{p_1}\lambda_{p_3}^2(\lambda_{p_3}-\lambda_{p_1})(t-T_{lag})-\lambda_{p_3}^2(\lambda_{p_3}-3\lambda_{p_1})) \\ & exp(\lambda_{p_1}(t-T_{lag}))+(\lambda_{p_3}-\lambda_{p_1})^3]. \end{aligned} \tag{S5}$$

For TSG, only both alleles are inactivated, TSG is inactivated [2]. Hence, the cells do not grow abnormally when only one one allele is inactivated. As a consequence, we need to consider the alternations in TSG. Inactivation of each allel of TSG has two ways, one way is mutation, another is a loss of heterozygosity (LOH). Studies showed that crypts carrying one of APC and KRAS alterations (that is the inactivation of APC and activation of KRAS) confers a selective growth advantage to the cell [3, 4]. Therefore, all possible orders for the alterations of three genes in Figure A-F can be summarized as the five-stage models with different clonal expansion of premalignant cells by the clonal expansion of premalignant cells, which is displayed as follows

(1) the five-stage model with $\lambda_{p_2} = \lambda_{p_1}$ and $\lambda_{p_4} = \lambda_{p_3}$ for the models (A1) and (B1);

(2) the five-stage model with $\lambda_{p_3} = \lambda_{p_2} = \lambda_{p_1}$ for the models (A3) and (B3);

(3) the five-stage model with $\lambda_{p_1} = 0$ and $\lambda_{p_3} = \lambda_{p_2}$ for the models (A4), (B4), (C1), (D2), (E1) and (F2);

(4) the five-stage model with $\lambda_{p_1} = 0$ and $\lambda_{p_4} = \lambda_{p_3}$ for the models (A2), (B2), (D1) and (F1);

(5) the five-stage model with $\lambda_{p_1} = 0$ and $\lambda_{p_2} = 0$ for the models (A5), (B5), (C2), (D3), (E2) and (F3).

Then, the hazard function for the case with $\lambda_{p_3} = \lambda_{p_2} = \lambda_{p_1}$ can be written as

$$\begin{aligned} h(t) \approx & \tfrac{N\mu_N\mu_{p_1}\mu_{p_2}\mu_{p_3}\mu_{p_4}}{\lambda_{p_1}^3\lambda_{p_4}(\lambda_{p_4}-\lambda_{p_1})^3}[\lambda_{p_1}^3 exp(\lambda_{p_4}(t-T_{lag}))-(\tfrac{\lambda_{p_1}^2\lambda_{p_4}(\lambda_{p_4}-\lambda_{p_1})^2}{2}(t-T_{lag})^2 \\ & +\lambda_{p_1}\lambda_{p_4}(\lambda_{p_4}-\lambda_{p_1})(2\lambda_{p_1}-\lambda_{p_4})(t-T_{lag})+3\lambda_{p_1}^2\lambda_{p_4}-3\lambda_{p_1}\lambda_{p_4}^2+\lambda_{p_4}^3) \\ & exp(\lambda_{p_1}(t-T_{lag}))+(\lambda_{p_4}-\lambda_{p_1})^3]. \end{aligned} \tag{S6}$$

The hazard function for the case with $\lambda_{p_1} = 0$ and $\lambda_{p_3} = \lambda_{p_2}$ can be written as

$$\begin{aligned} h(t) \approx & \tfrac{N\mu_N\mu_{p_1}\mu_{p_2}\mu_{p_3}\mu_{p_4}}{\lambda_{p_2}^3\lambda_{p_4}^2(\lambda_{p_4}-\lambda_{p_2})^2}[(\lambda_{p_2}\lambda_{p_4}(\lambda_{p_4}-\lambda_{p_2})(2\lambda_{p_4}-\lambda_{p_2})(t-T_{lag})+\lambda_{p_2}^3) \\ & exp(\lambda_{p_4}(t-T_{lag}))-(\lambda_{p_2}\lambda_{p_4}^2(\lambda_{p_4}-\lambda_{p_2})(t-T_{lag})+\lambda_{p_4}^2(3\lambda_{p_2}-2\lambda_{p_4})) \\ & exp(\lambda_{p_2}(t-T_{lag}))-(\lambda_{p_2}\lambda_{p_4}(\lambda_{p_4}-\lambda_{p_2})^2(t-T_{lag}) \\ & +(\lambda_{p_4}-\lambda_{p_2})^2(2\lambda_{p_4}+\lambda_{p_2}))]. \end{aligned} \tag{S7}$$

For the case with $\lambda_{p_1} = 0$ and $\lambda_{p_4} = \lambda_{p_3}$, the hazard function yields

$$\begin{aligned} h(t) \approx & \tfrac{N\mu_N\mu_{p_1}\mu_{p_2}\mu_{p_3}\mu_{p_4}}{\lambda_{p_2}^2\lambda_{p_3}^4(\lambda_{p_3}-\lambda_{p_2})^2}[(\lambda_{p_2}^2\lambda_{p_3}^2(\lambda_{p_3}-\lambda_{p_2})^2(t-T_{lag})+\lambda_{p_2}\lambda_{p_3}(2\lambda_{p_2}^2-3\lambda_{p_2}\lambda_{p_3})) \\ & exp(\lambda_{p_3}(t-T_{lag}))+\lambda_{p_3}^4 exp(\lambda_{p_2}(t-T_{lag}))-(\lambda_{p_2}\lambda_{p_3}^2(\lambda_{p_3}-\lambda_{p_2})^2(t-T_{lag}) \\ & +\lambda_{p_3}^4+2\lambda_{p_2}^3\lambda_{p_3}-3\lambda_{p_2}^2\lambda_{p_3}^2)]. \end{aligned} \tag{S8}$$

The hazard function for the case with $\lambda_{p_1} = 0$ and $\lambda_{p_2} = 0$ can given by

$$\begin{aligned} h(t) \approx & \tfrac{N\mu_N\mu_{p_1}\mu_{p_2}\mu_{p_3}\mu_{p_4}}{\lambda_{p_3}^3\lambda_{p_4}^3(\lambda_{p_4}-\lambda_{p_3})}[\lambda_{p_3}^3 exp(\lambda_{p_4}(t-T_{lag}))-\lambda_{p_4}^3 exp(\lambda_{p_3}(t-T_{lag})) \\ & +\tfrac{\lambda_{p_3}^2\lambda_{p_4}^2(\lambda_{p_4}-\lambda_{p_3})}{2}(t-T_{lag})^2+\lambda_{p_3}\lambda_{p_4}(\lambda_{p_4}^2-\lambda_{p_3}^2)(t-T_{lag}) \\ & +(\lambda_{p_4}^3-\lambda_{p_3}^3)]. \end{aligned} \tag{S9}$$

The derivation of Kolmogorov backward equations

Here, we mainly give the differential equation for Φ_1, the derivations of Kolmogorov backward equation for other probability generating functions can be obtained similarly. We consider the $\Phi_1(x_1, x_2, x_3, x_4, y; 0, t+\Delta)$, where Δ is very small increment in time. By the Chapman-Kolmogorov equations, the $\Phi_1(x_1, x_2, x_3, x_4, y; 0, t+\Delta)$ can be written as

$$\begin{aligned}\Phi_1(x_1, x_2, x_3, x_4, y; 0, t+\Delta) = & \sum_{i_1,i_2,i_3,i_4,j} \sum_{n_1,n_2,n_3,n_4,m} prob\Big\{P_1(t+\Delta) = i_1, P_2(t+\Delta) = i_2, \\ & P_3(t+\Delta) = i_3, P_4(t+\Delta) = i_4, M(t+\Delta) = j | P_1(\Delta) = n_1, P_2(\Delta) = n_2, \\ & P_3(\Delta) = n_3, P_4(\Delta) = n_4, M(\Delta) = m\Big\} prob\Big\{P_1(\Delta) = n_1, \\ & P_2(\Delta) = n_2, P_3(\Delta) = n_3, P_4(\Delta) = n_4, M(\Delta) = m | P_1(0) = 1, \\ & P_2(0) = 0, P_3(0) = 0, P_4(0) = 0, M(0) = 0\Big\} x_1^{i_1} x_2^{i_2} x_3^{i_3} x_4^{i_4} y^j. \qquad \text{(S10)}\end{aligned}$$

It is well known that at most one event occurs in a sufficiently small amount of time. Thus the probabilities of the events that can occur in $[0, \Delta]$ are approximately $\alpha_{P_i}\Delta$ $(i = 1, 2, 3, 4)$ for the birth of a state P_i cell, $\beta_{P_i}\Delta$ for the death of a state P_i cell, $\mu_{P_i}\Delta$ for the transformation from state P_i to P_{i+1} (from state P_4 to M if $i = 4$) and $1 - \alpha_{P_i}\Delta - \beta_{P_i}\Delta - \mu_{P_i}\Delta$ for no change in the state P_i, respectively. Then, we get

$$\begin{aligned}\Phi_1(x_1, x_2, x_3, x_4, y; 0, t+\Delta) = & \sum_{i_1,i_2,i_3,i_4,j} \Bigg\{ \alpha_{p_1}\Delta prob\Big\{P_1(t+\Delta) = i_1, P_2(t+\Delta) = i_2, \\ & P_3(t+\Delta) = i_3, P_4(t+\Delta) = i_4, M(t+\Delta) = j | P_1(\Delta) = 2, \\ & P_2(\Delta) = 0, P_3(\Delta) = 0, P_4(\Delta) = 0, M(\Delta) = 0\Big\} \\ & +\beta_{p_1}\Delta prob\Big\{P_1(t+\Delta) = i_1, P_2(t+\Delta) = i_2, \\ & P_3(t+\Delta) = i_3, P_4(t+\Delta) = i_4, M(t+\Delta) = j | P_1(\Delta) = 0, \\ & P_2(\Delta) = 0, P_3(\Delta) = 0, P_4(\Delta) = 0, M(\Delta) = 0\Big\} \\ & +\mu_{p_1}\Delta prob\Big\{P_1(t+\Delta) = i_1, P_2(t+\Delta) = i_2, P_3(t+\Delta) = i_3, \\ & P_4(t+\Delta) = i_4, M(t+\Delta) = l | P_1(\Delta) = 1, P_2(\Delta) = 1, \\ & P_3(\Delta) = 0, P_4(\Delta) = 0, M(\Delta) = 0\Big\} \\ & +(1 - \alpha_{p_1}\Delta - \beta_{p_1}\Delta - \mu_{p_1}\Delta) prob\Big\{P_1(t+\Delta) = i_1, P_2(t+\Delta) = i_2, \\ & P_3(t+\Delta) = i_3, P_4(t+\Delta) = i_4, M(t+\Delta) = j | P_1(\Delta) = 1, \\ & P_2(\Delta) = 0, P_3(\Delta) = 0, P_4(\Delta) = 0, M(\Delta) = 0\Big\} \\ & \Bigg\} x_1^{i_1} x_2^{i_2} x_3^{i_3} x_4^{i_4} y^j. \qquad \text{(S11)}\end{aligned}$$

We let $\Phi_1(t+\Delta)$ to denote the $\Phi_1(x_1, x_2, x_3, x_4, y; 0, t+\Delta)$. By the definition for $\Phi_1(t)$, Markov property and relationship formula (3) in reference [5], the formula (S11) can be written as

$$\begin{aligned}\Phi_1(t+\Delta) = & \alpha_{p_1}\Delta\Phi_1^2(t) + \beta_{p_1}\Delta + \mu_{p_1}\Delta\Phi_1(t)\Phi_2(t) \\ & +(1 - \alpha_{p_1}\Delta - \beta_{p_1}\Delta - \mu_{p_1}\Delta)\Phi_1(t). \qquad \text{(S12)}\end{aligned}$$

Subtracting $\Phi_1(t)$ from both sides of above formula and divide by Δ, then we obtain

$$\frac{d\Phi_1}{dt}(t) = -(\alpha_{p_1} + \beta_{p_1} + \mu_{p_1})\Phi_1(t) + \alpha_{p_1}\Phi_1^2(t) + \mu_{p_1}\Phi_1(t)\Phi_2(t) + \beta_{p_1}. \tag{S13}$$

$\frac{d\Phi_k}{dt}(t)$ and $\frac{d\Psi}{dt}(t)$ are obtained similarly, where $k = 2, 3, 4$.

The expected number of mutant cell

For the orders of genetic mutations with $KRAS - APC - TP53$, $APC - TP53 - KRAS$ and $APC - KRAS - TP53$ that can explain the incidence rate of colorectal cancer, the number of premalignant cells with only $KRAS^+$, $APC^{-/-}$, $KRAS^+APC^{-/-}$, $APC^{-/-}KRAS^+$ and $APC^{-/-}TP53^{-/-}$ can be calculated by the equations (S4). The results are as follows

$$\begin{cases} E[n_{KRAS^+}(t)] = \frac{N\mu_N}{\lambda_{p_1}}(e^{\lambda_{p_1}t} - 1), \\ E[n_{APC^{-/-}}(t)] = \frac{N\mu_N\mu_{p_1}}{\lambda_{p_2}^2}(e^{\lambda_{p_2}t} - \lambda_{p_2}t - 1), \\ E[n_{KRAS^+APC^{-/-}}(t)] = N\mu_N\mu_{p_1}\mu_{p_2}[\frac{e^{\lambda_{p_3}t}}{\lambda_{p_3}(\lambda_{p_3}-\lambda_{p_1})^2} - \frac{1}{\lambda_{p_1}(\lambda_{p_3}-\lambda_{p_1})}(t - \frac{1}{\lambda_{p_1}} \\ \qquad + \frac{1}{\lambda_{p_3}-\lambda_{p_1}})e^{\lambda_{p_1}t} - \frac{1}{\lambda_{p_1}^2\lambda_{p_3}}], \\ E[n_{APC^{-/-}KRAS^+}(t)] = \frac{N\mu_N\mu_{p_1}\mu_{p_2}}{\lambda_{p_2}^2\lambda_{p_3}^2(\lambda_{p_3}-\lambda_{p_2})}[\lambda_{p_2}^2 e^{\lambda_{p_3}t} - \lambda_{p_3}^2 e^{\lambda_{p_2}t} + \lambda_{p_2}\lambda_{p_3}(\lambda_{p_3} \\ \qquad - \lambda_{p_2})t + \lambda_{p_3}^2 - \lambda_{p_2}^2], \\ E[n_{APC^{-/-}TP53^{-/-}}(t)] = \frac{N\mu_N\mu_{p_1}\mu_{p_2}\mu_{p_3}}{\lambda_{p_2}^3\lambda_{p_4}^2(\lambda_{p_4}-\lambda_{p_2})^2}[(\lambda_{p_2}\lambda_{p_4}(\lambda_{p_4} - \lambda_{p_2})(2\lambda_{p_4} - \lambda_{p_2})t \\ \qquad + \lambda_{p_2}^3)e^{\lambda_{p_4}t} - (\lambda_{p_2}\lambda_{p_4}^2(\lambda_{p_4} - \lambda_{p_2})t + \lambda_{p_4}^2(3\lambda_{p_2} - \\ \qquad 2\lambda_{p_4}))e^{\lambda_{p_2}t} - (\lambda_{p_2}\lambda_{p_4}(\lambda_{p_4} - \lambda_{p_2})^2 t \\ \qquad + (\lambda_{p_4} - \lambda_{p_2})^2(2\lambda_{p_4} + \lambda_{p_2}))], \\ E[n_{APC^{-/-}TP53^{-/-}}(t)] = \frac{N\mu_N\mu_{p_1}\mu_{p_2}\mu_{p_3}}{\lambda_{p_3}^3\lambda_{p_4}^3(\lambda_{p_4}-\lambda_{p_3})}[\lambda_{p_3}^3 e^{\lambda_{p_4}t} - \lambda_{p_4}^3 e^{\lambda_{p_3}t} \\ \qquad + \frac{\lambda_{p_3}^2\lambda_{p_4}^2(\lambda_{p_4}-\lambda_{p_3})}{2}t^2 + \lambda_{p_3}\lambda_{p_4}(\lambda_{p_4}^2 \\ \qquad - \lambda_{p_3}^2)t + (\lambda_{p_4}^3 - \lambda_{p_3}^3)], \end{cases} \tag{S14}$$

References

[1] Moolgavkar SH, Dewanji A, Venzon DJ. A Stochastic Two-Stage Model for Cancer Risk Assessment. I. The Hazard Function and the Probability of Tumor. Risk Anal. 1988;8: 383–392.

[2] Vogelstein B, Kinzler KW. Cancer genes and the pathways they control. Nat Med. 2004;10: 789–799.

[3] Lamlum H, Papadopoulou A, Ilyas M, Rowan A, Gillet C, Hanby A, et al. APC mutations are sufficient for the growth of early colorectal adenomas. Proc Natl Acad Sci U S A. 2000;97: 2225–2228.

[4] Snippert HJ, Schepers AG, van Es JH, Simons BD, Clevers H. Biased competition between Lgr5 intestinal stem cells driven by oncogenic mutation induces clonal expansion. EMBO Rep. 2014;15: 62–69.

[5] Portier CJ, Sherman CKA. Calculating tumor incidence rates in stochastic models of carcinogenesis. Math Biosci. 1996;135(2): 129–146.
